# Effect of Post-Heat Treatment on the Mechanical and Residual Stress Behavior of Pulsed Wave S316L Fabricated by Directed Energy Deposition

**DOI:** 10.3390/s24237457

**Published:** 2024-11-22

**Authors:** Zhou Yan, Jia Guo, Xi Zou

**Affiliations:** 1 School of Information Engineering, Hubei University of Economics, Wuhan 430205, China; 2 Hubei Key Laboratory of Digital Finance Innovation, Hubei University of Economics, Wuhan 430205, China; 3 Hubei Internet Finance Information Engineering Technology Research Center, Hubei University of Economics, Wuhan 430205, China; 4 Faculty of Computer and Information Sciences, Hosei University, Tokyo 102-8160, Japan; 5 Mechanical and Electrical Engineering College, Hunan University of Science and Technology, Xiangtan 411201, China; 103075@hnust.edu.cn

**Keywords:** DED additive manufacturing, microstructure, mechanical properties, residual stress

## Abstract

The influence of annealing at various temperatures on the phase stability and microstructure of pulsed-wave laser mode (PW) 316L stainless steel fabricated via Directed Energy Deposition (DED) was systematically investigated. The microstructural alterations resulting from heat treatment were examined to clarify their influence on the mechanical properties of the specimens subjected to tensile loading. The results showed that cell size increased with annealing temperature, with the cellular microstructure disappearing at higher temperatures (T ≥ 1000 °C). A decrease in the mechanical strength of the specimens was observed as annealing temperature increased. Additionally, the influence of different laser pulse frequencies and duty cycles on residual stresses was examined, revealing that moderate laser frequencies and duty cycles effectively reduced residual stress levels.

## 1. Introduction

Additive manufacturing (AM) emerged some forty years ago as a technique for making components using a layer-by-layer process. Additive manufacturing offers notable benefits compared to conventional manufacturing techniques, such as minimized material waste and post-processing requirements, enhanced design flexibility, and reduced expenses, particularly for the small- to medium-batch production of performance-critical components [1]. Although metal additive manufacturing (AM) has many acknowledged benefits, there are still not many effective applications to date. This is due to the fact that technical obstacles still prevent AM from reaching its full potential [2,3,4,5]. Firstly, steel’s microstructure—which is influenced by phase transitions, precipitation, and recrystallization—has a major impact on its mechanical characteristics. Austenitic stainless steels’ hardness and strength are unaffected by heat treatment [6,7], as strengthening mostly depends on the steels’ phase transition properties, particularly the austenite–martensite transformation [8].

Secondly, the residual stresses (RSs) generated by nonuniform thermal gradients during rapid heating and cooling cycles in additive manufacturing methods present a considerable difficulty. Thermally produced residual stresses and the consequent distortions are significant obstacles to the widespread implementation of additive manufacturing technology [5,9,10]. Research has suggested that AM-produced components can exhibit very high RS levels [10,11]. For instance, as-built H13 steel samples have been found to have RS values in the range of 940–1420 MPa, significantly higher than their yield strength of 1650 MPa [12]. The impact of RS depends on its distribution and whether it is compressive or tensile. Compressive RS near the surface of a component can improve stress-corrosion cracking resistance and fatigue strength, whereas tensile RS increases the likelihood of stress-corrosion cracking, potentially leading to premature component failure [13,14]. In metal AM processes, RS can not only negatively affect the mechanical performance of components but also reduce geometric precision [15,16].

A multitude of studies has been undertaken on residual stress (RS) in additively manufactured items, focusing on the causes of RS generation, the impact of process parameters on RS development, RS simulation, and strategies for mitigating RS through in situ and post-processing approaches. A multitude of reviews have synthesized the literature on residual stress in metal additive manufacturing. Megahed et al. [16] and Srivastava et al. [17] assembled sophisticated computational techniques for RS prediction in additive manufacturing processes. Bartlett and Li [18] investigated the evolution of residual stress (RS) in powder bed fusion (PBF) processes, emphasizing modeling methodologies and the impact of process factors. Carpenter and Tabei [19] examined modeling and measuring methodologies for RS in AM, along with measures to mitigate and minimize RS-induced distortions. Chen et al. [20] presented a comprehensive overview of residual stress generation, parameter dependency, prediction, and control in metal additive manufacturing processes, whereas Fang et al. [21] concentrated on residual stress research specifically within laser powder bed fusion techniques. Notwithstanding these endeavors, a deficiency of thorough investigations of RS in the two principal metal additive manufacturing methods (i.e., powder bed fusion (PBF) and Directed Energy Deposition (DED)) persists. Furthermore, a thorough assessment of the current literature is essential to enhance the comprehension of the problems and prospects for future study in this swiftly evolving domain. The pulsed-wave (PW) laser mode, unlike the continuous-wave (CW) laser mode, employs intermittent heating and cooling cycles, leading to less heat accumulation and enhanced cooling speeds. This cyclic heating method results in a finer microstructure [22], but necessarily introduces various residual stresses. R.J. Moat [23] investigated the influence of laser pulse width and duty cycle on the residual stress values and their distribution in nickel-based superalloys during multi-channel laser direct metal deposition. Fischer’s study looked in depth at the effects of a pulsed laser on additively built 316L stainless steel using laser powder bed fusion (L-PBF). It proved that dense components may be generated across a wide range of parameters using pulsed-laser L-PBF [24]. Zhang investigated a laser welding method for dissimilar materials of 316L stainless steel (316L SS) and polylactic acid (PLA), developed to assess the process factors that have a significant impact on joint quality [25]. Using the response surface methodology (RSM), Yesudhasan finds that optimizing the welding speed at 120 mm/min, voltage at 15 volts, amperage at 140 amperes, and gas flow rate at 15 L per minute significantly improves weld strength and quality [26]. Zou demonstrates that the pulsed-wave (PW) laser mode reduced residual stress by around 75% during the direct laser deposition technique. The decrease in residual stress in the PW laser mode is due to a lower temperature gradient and stress release [27]. Yan demonstrates how the lowering of the temperature gradient and stress release contribute to the reduction and homogeneity of residual stress in the PW laser mode [28].

This work primarily examines the effects of heat treatment on the microstructure, mechanical characteristics, and residual stress fields produced by PW modes at different heat treatment temperatures. We utilized the contour method to delineate the longitudinal residual stress across the substrate’s depth induced by Laser Additive Manufacturing.

The contour method, an innovative technique for assessing residual stress, records the deformation of a severed part resulting from the release of residual stress perpendicular to the cutting plane. The outcomes from this method, a widely employed nondestructive technique, were collected from uniform positions along the substrate centerline. Furthermore, the vertical residual stress in the longitudinal direction (aligned with the laser scanning direction) was quantified utilizing the contour approach.

## 2. Materials and Procedures

**Figure 1:**
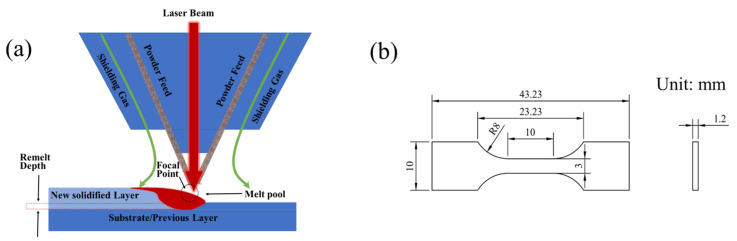
(**a**) A visual representation of the DED experiment. (**b**) The dimensions of the under-tension sample.

**Figure 2:**
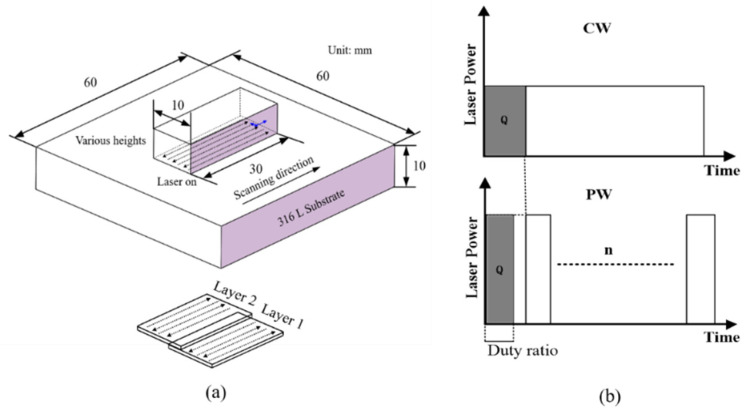
(**a**) A block for direct laser deposition, (**b**) a diagram showing the architecture of CW and PW lasers.

The substrate material employed in this experiment was 316L stainless steel with dimensions of 60 × 60 × 10 mm. The deposition powder utilized was 316L stainless steel, with its composition detailed in Table 1. The illustrated process flow for additive manufacturing is shown in Figure 1a. A dog-bone-shaped specimen was prepared for tensile testing, as illustrated in Figure 1b. The loading rate was established at 0.1 mm/min, with an unloading rate of 30 N/s and an unloading force of 30 N. The tensile direction was oriented perpendicular to the building direction. Before the trials, the powder underwent processing in a vacuum drying chamber, where the temperature was elevated to 120 °C within 20 min to guarantee thorough moisture elimination. Samples were fabricated with a laser direct deposition system, comprising a YLS-4000-CL fiber laser (wavelength: 1070 nm), an IRB2400/16 six-axis robotic apparatus, a high-precision powder feeder, a coaxial nozzle, and an atmosphere control system, among other elements. Figure 2a presents a schematic design of the system.

To examine the influence of laser modes (Continuous Wave and Pulsed Wave) on residual stress at an identical peak power, a laser power of 500 W was employed for both modes. For the PW mode, the pulse cycle time was set to 0.1 s (10 Hz pulse frequency) with a duty ratio of 75%, as illustrated in Figure 2b (pulse chart). Duty ratios of 33.3%, 50%, 66.7%, and 75% were tested to maintain consistent energy output under different pulse frequencies (5, 20, 100, and 500 Hz) while keeping all other conditions constant. The process parameters are specified in Table 2.

**Figure 3:**
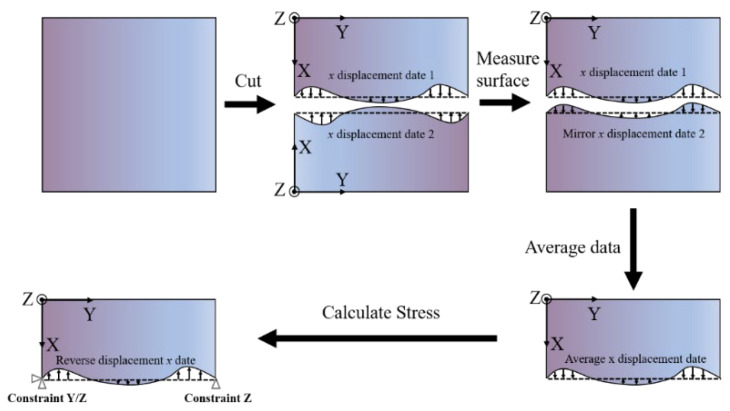
Diagram depicting the sample dimensions. Measurement sites: contour on the cut surface.

Each sample consisted of 20 layers, with a layer height increment of 0.2 mm, and was constructed using 12 single tracks with a hatch spacing of 1 mm, resulting in deposits 12 mm wide, 30 mm long, and varying in height. After deposition, the material was separated from the substrate and divided into two sections to measure residual stress using the contour method (CM). The CM was employed to produce a cross-sectional (2D) map of the residual stresses perpendicular to the cross-section, while XRD was used to measure and validate the CM results, specifically the longitudinal residual stress along the depth direction from the substrate centerline, as shown in Figure 3. The second group of samples had their vertical residual stress measured using the CM. Other processing parameters included an argon protective gas flow rate of 8 L/min and a spot diameter of 1 mm, with all scanning paths following a one-way scanning pattern. After the clad bulk was cut using electro-discharge machining (EDM), samples were prepared for microstructure analysis. Electron scanning microscopy (ESM) and optical microscopy (OM) used a solution of 30 mL HCl and 10 mL HNO3 (SEM, JSM-5600LV, JEOL) for mounting, grinding, polishing, and etching. A scanning electron microscope (SEM) coupled with an energy-dispersive spectrometer (EDS) was used to examine the composition. Electron backscatter diffraction (EBSD) was performed by electropolishing specimens in a 10% perchlorate alcohol solution for 30 s at 20 V.

The contour method, a recognized approach for delineating residual stresses, generates a two-dimensional map of residual stresses perpendicular to the cut surface. It operates based on the Elastic Superposition Principle, where the displacements of the cut surface (the surface contour) are compared to an assumed flat surface. This method allows residual stresses to be relieved as the material is slit. Subsequently, a finite element model is applied to impose the inverse of the observed contour displacements. This process involves three key steps: first, cutting the material; second, measuring the surface contour; and third, calculating the resulting stresses. A detailed explanation of the method can be found in refs. [22,23].

### 2.1. Making Cut

**Figure 4:**
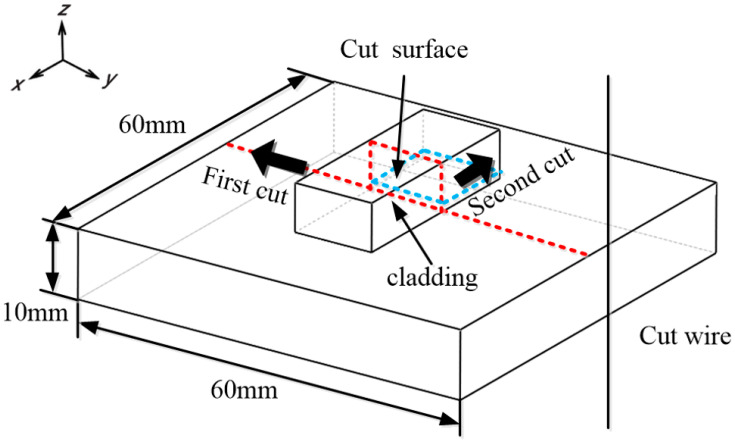
Schematic diagram of wire cutting.

The optimal method for producing the cut must meet the following criteria: (1) create a straight cut; (2) avoid removing any additional material from the previously cut surfaces; and (3) ensure no additional stresses are introduced during the cutting process. The preferred technology for this is wire electric discharge machining (wire EDM), which uses 100 um diameter brass wires to split each deposit. The cutting procedure involves two steps: first, the complete sample is bisected at its midpoint; thereafter, the deposited material is excised 1 mm above the root, as illustrated in Figure 4. The specimen is securely fastened on both sides to prevent any movement during cutting. The “skim cut” method is selected to minimize any stresses induced by the cutting process [22,23].

### 2.2. Measuring the Surface Contour

After splitting the deposit into two sections, the normal-direction displacements of each part’s surface were measured. A coordinate measuring machine (CMM) is among the most straightforward and generally available techniques for assessing the contour of a newly cut surface. In this research, a Hexagon Metrology coordinate measuring machine (EXPLORER-10.21.08) was used to measure the surface contours. The first cut was utilized to accurately capture the residual stresses in the deposited materials.

**Figure 5:**
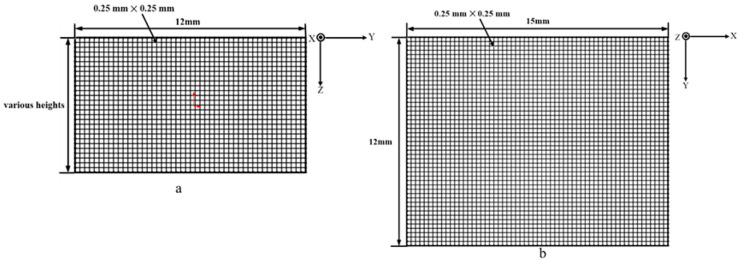
Schematic of CMM measuring points: (**a**) first cut and (**b**) second cut.

Figure 5a illustrates a uniform distribution of encrypted inspection points applied across the cross-section. The measurement spacing between two inspection points was set at 0.25 mm, and the overall measurement area was selected based on the actual cross-sectional area, as the height varied due to different process conditions. For the second cut, the measurement area was adjusted to 12 mm × 15 mm, with the same measurement spacing of 0.25 mm, as shown in Figure 5b.

### 2.3. Data Processing

The initial step in data processing involved aligning both sides of the ‘cut data’ to the same coordinate frame, ensuring that the two cut surfaces appeared as mirror images. To achieve this, we mirrored the data from one surface and then averaged it with the other. This approach helps to minimize deviations caused by potential errors during wire EDM operations, such as surface slanting. Noise and surface roughness were further reduced by applying a smoothing technique to the averaged data [23]. Although the measured data may include minor cutting-edge errors, these can be considered negligible.

### 2.4. Calculating Stress

ANSYS 18.0 was utilized to develop a 3D finite element (FE) model for stress analysis on one half of the substrate. The material was characterized as homogenous, isotropic, and linearly elastic, possessing a Young’s modulus of 200 GPa as well as a Poisson’s ratio of 0.3. The sample was constructed using 8-node brick elements (element type 185) and meshed with hexahedral components. After averaging and filtering the displacement data, the final displacements, with the opposite sign, were applied to the FE model’s surface as displacement boundary conditions. Furthermore, three-point boundary constraints were incorporated into the model to restrict rigid-body motion.

### 2.5. LDS Displacement Transducer

**Figure 6:**
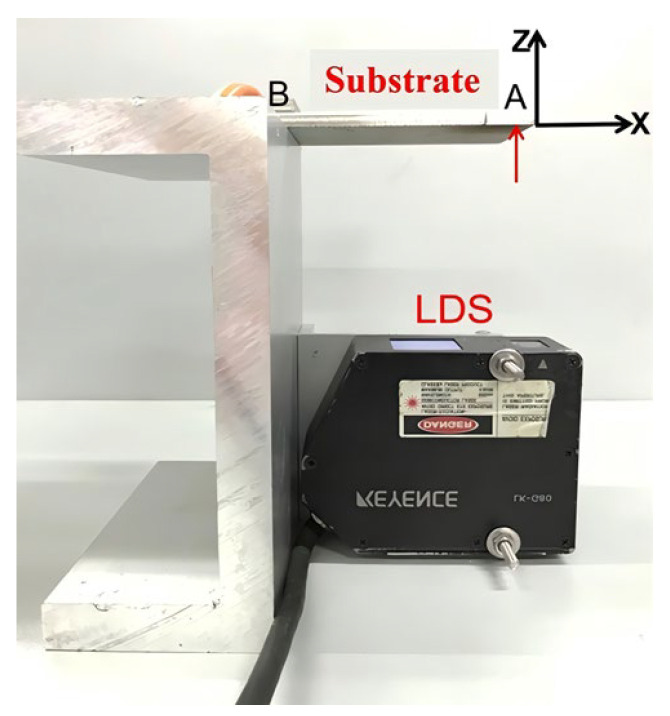
LDS displacement transducer.

In this section, the effect of different scan paths on the substrate deformation and the effect of different scan starting points on the substrate deformation are studied, as illustrated in Figure 6. Point A: the measurement point; print length: 80 mm; layer: 15; and starting point print direction on the free end point A: two-way print. Base board suspended length: 80 mm; 316 base board: 140 mm× 40 mm × 3 mm; powder: F3000; send powder quantity: 8 g/mint; power: 500 W; and speed: 8 mm/s. Point B: the terminus measurement point as shown Figure 6.

### 2.6. Thermomechanical Simulations

**Figure 7:**
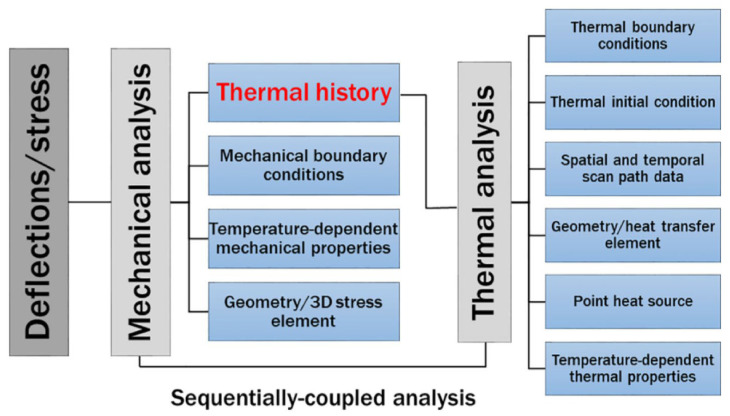
The block diagram summarizes the model flow principle using the indirect thermal coupling model analysis approach.

The finite element method (FEM) was utilized to model the development and dispersion of the temperature field during the Directed Energy Deposition (DED) of austenitic stainless steels. The birth–death element technique was employed in the simulation phase, during which all deposit components remained dormant prior to the commencement of deposition. Elements were activated following the deposition sequence at each time step through an octree-based search technique as shown in Figure 7.

Modeling parameters pertinent to austenitic stainless steels, including thermal properties (e.g., thermal conductivity, specific heat capacity, and coefficient of thermal expansion), temperature-dependent mechanical properties (e.g., Young’s modulus), and modeling dimensions, boundary conditions, and governing equations were obtained from our prior research [24]. A succinct overview of the model is presented herein. 

The equation that governs transient heat conduction in an element subjected to a volumetric heat source during the DED process is articulated as follows:

(1) $$Q(x,t)-\nabla \cdot q(x,t)=\rho C_{p}\frac{dT}{dt}$$

where Q represents density, $C_{p}$ denotes the temperature-dependent specific heat capacity, T signifies temperature, t indicates time, and q refers to the conductive heat flow through the material. The average volumetric heat source model for a laser beam is

(2) $$Q_{\left(x,t\right)}=\frac{\eta P}{V}$$

where η represents alloy laser absorption, P signifies laser power, and V defines the volume affected by the heat source.

The DED process is believed to encompass three forms of heat transmission: conductive heat transfer (*q*), radiative heat transfer ($q_{rad}$), and convective heat transfer ($q_{conv}$). The three modes of heat transmission, in sequence, adhere to Fourier’s law, Stefan–Boltzmann’s law, and Newton’s law of convective cooling.

(3) q=−k∇T

(4) $$q_{rad}=\varepsilon \sigma_{b}\left(T_{S}^{4}-T_{\infty }^{4}\right)$$

(5) $$q_{conv}=h\left(T_{S}-T_{\infty }\right)$$

where *k* represents the temperature-dependent thermal conductivity; *ε* denotes the surface emissivity; $\sigma_{b}$ is the Stefan–Boltzmann constant; $T_{S}$ and $T_{\infty }$ signify the surface temperature of the deposit and the ambient temperature, respectively; and *h* indicates the convective heat transfer coefficient.

The equation for the equilibrium of managing mechanical stress can be expressed as follows:

(6) ∇⋅σ=0

where σ represents the third-order stress tensor. The mechanical constitutive law is delineated as follows:

(7) $$\sigma =C\varepsilon_{e}$$

where C represents the fourth-order stiffness tensor and $\varepsilon_{e}$ signifies the elastic strain:

(8) $$\varepsilon_{e}=\varepsilon -\varepsilon_{p}-\varepsilon_{T}$$

The hatch spacing (h) and powder layer thickness (t) were maintained constant, while the laser power (P) and scanning speed (υ) were varied, resulting in different volumetric energy densities ($E_{v}$) determined according to Equation (1) [25].

(9) $$E_{v}=\frac{P}{vht}\left(\frac{J}{mm^{3}}\right)$$

## 3. Results and Discussion

### 3.1. Characterization of Materials

#### 3.1.1. XRD and Electron Backscatter Diffraction (EBSD) Measurements

**Figure 8:**
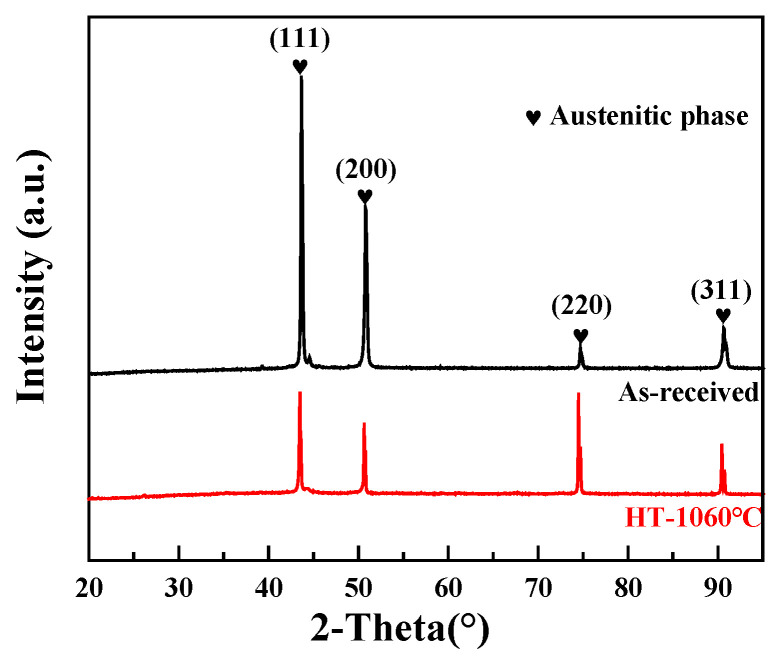
Experimental procedure schematic for DED XRD metal and as-received sample spectra.

**Figure 9:**
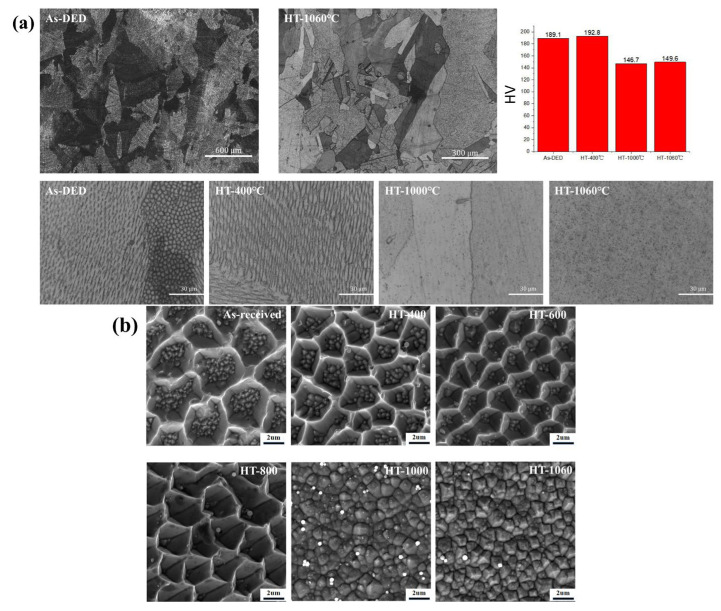
(**a**) Images taken of the solidification cell of both the untreated and heated samples using an optical microscope, and (**b**) enlarged SEM images under different heat treatment conditions.

**Figure 10:**
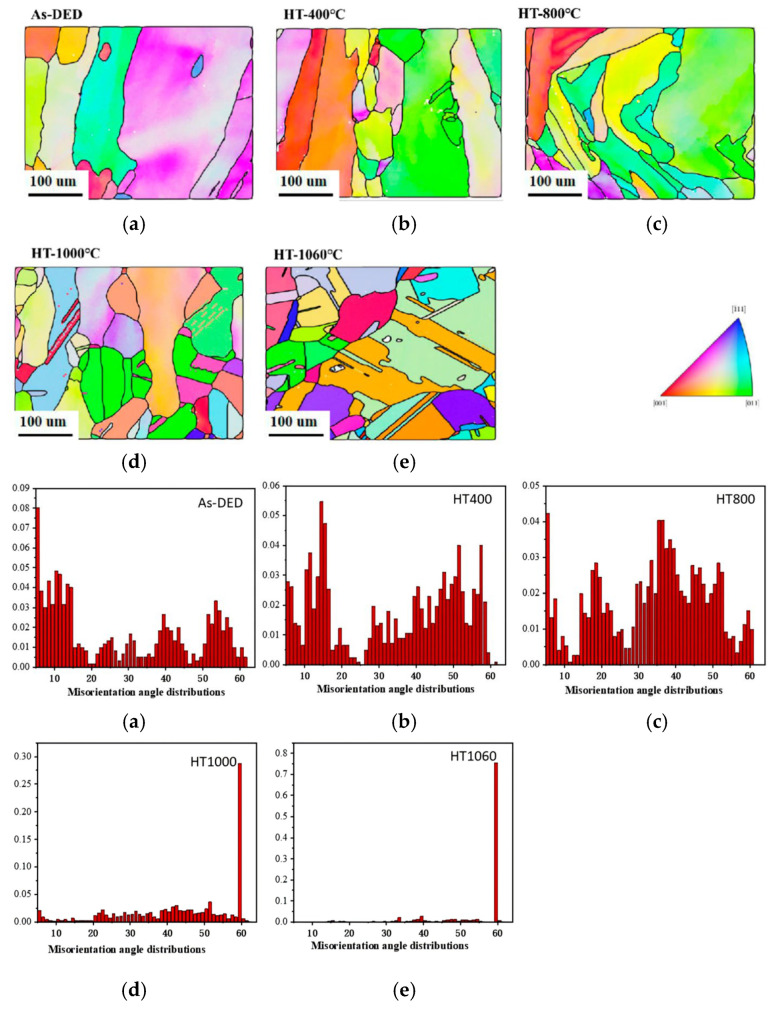
Grain maps and misorientation angle distributions of 316L stainless steel evaluated with EBSD for (**a**) DED samples as well as specimens annealing at (**b**) 400 °C, (**c**) 800 °C, (**d**) 1000 °C, and (**e**) 1060 °C.

In Figure 8, we can see the XRD data for both the as-received and HT samples, red text color is the XRD data line at temperature 1060 of heat treatment, showing the strongest ferrite peaks typically observed at 44.7° and 65°. Figure 9a shows images taken of the solidification cell of both the untreated and heated samples using a light microscope, and Figure 9b shows enlarged SEM images under different heat treatment conditions. The observed decrease in hardness is likely since the cellular structure has vanished [26]. The development of a heterogeneous cell structure throughout heat treatment was viewed using SEM (Figure 9b). Solidification cells were implanted inside every enormous individual grain. The solidification cell’s scale changed in the grain owing to the development pattern and temperature gradient effect. It can be seen that the solidification cell size’s diameter primarily ranged from 2.2 ± 0.07 um to 2.0 ± 0.3 um. When the temperature rises to 1000 degrees Celsius, coagulated cells appear homogenized, and the size is 0 um. Our previous work [29] demonstrated the trapping of Cr and Mo elements between solidification cells, which is consistent with the segregation simulation study of the SLM solution by T. Pinomaa et al. on the rapidly solidified 316L stainless steel. As the capture of solutes and the degree of supercooling increase, the degree of segregation also decreases with the increase in solidification rate. The scanning rate and cooling rate of the DED process are lower than those of the SLM process, and it is generally reported that there is a larger cell size [20]. With the increase in heat treatment temperature, the diffusion ability of solidified cells to Cr and Mo elements decreases and gradually ablates. Figure 10a also includes the Inverse Pole Figure (IPF), highlighting the differences in grain size under various heat treatment conditions. The average grain sizes of the samples were 17.3 um, 18.1 um, 29.0 um, and homogenized, respectively. The hardness values for the four samples were measured as 189.1 Hv, 192.8 Hv, 146.7 Hv, and 146.9 Hv.

According to the XRD patterns shown in Figure 8, both the as-DED specimens and the samples that were heat-treated at 1060 °C displayed a structure of single-phase austenite. This is because the cooling rate and chemical composition of the alloy, especially the Cr/Ni ratio, have a significant role in the phase change of austenitic stainless steel. The orientation and morphology of the grains were examined using electron backscatter diffraction (EBSD) measurements both before and after heat treatment. On the SD-TD plane, Figure 10 displays the grain size, distribution, and form. Grain diameters averaged 45 ± 1 um for the as-DED and 400 °C samples, while lower annealing temperatures led to smaller and more equiaxed grains (Figure 10a,b). Figure 10c–e show that the grains grew bigger and more equiaxed as the annealing temperature rose. As a result of annealing at 800 °C, 1000 °C, and 1060 °C, the average grain sizes rose to 65 ± 8 um, 88 ± 5 um, and 102 ± 3 um, respectively. At temperatures up to 1000 °C, the data show that the cellular structure is preserved.

#### 3.1.2. Dislocation Density

**Figure 11:**
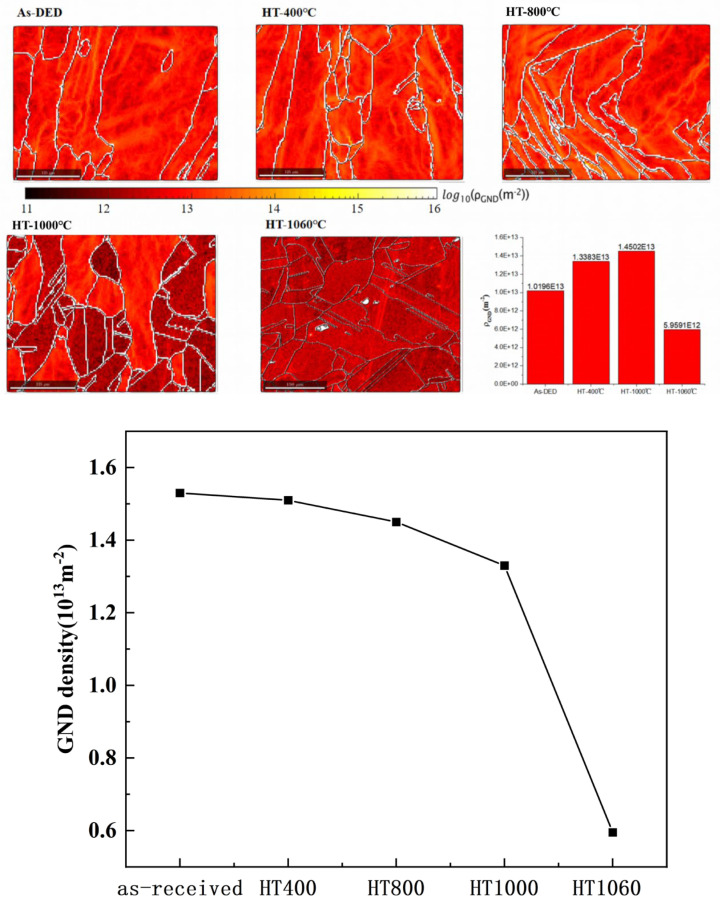
GND density mapping and values after different heat treatment temperatures.

The concept of geometrically necessary dislocation (GND) arrays has been a subject of extensive discussion in the literature. Our view is that the geometrically necessary dislocations represent an extra storage of dislocations required to accommodate the lattice curvature that arises whenever there is a nonuniform plastic deformation. Several papers have demonstrated that GND arrays can be used to estimate dislocation density using EBSD information-gauged kernel-averaged misorientation (KAM). Equation (1) follows from the strain slope theory and accounts for the density of geometrically necessary dislocations.

$$\rho^{GND}=\frac{2\theta }{ub}$$

where $\rho^{GND}$ denotes the GND density, u refers to the scanning stage (1 um), the local misorientation is indicated by q, and b represents the Burger’s vector. Utilizing this knowledge, tailored MATLAB, 14.0 content was implemented, and GND density is presented the equivalent positions of the KAM, as depicted in Figure 11. The average $\rho^{GND}$ of the as-received, HT400, HT800, HT1000, and HT1060 was 1.53 × 10^13^ m^−2^, 1.51 × 10^13^ m^−2^, 1.45 × 10^13^ m^−2^, 1.33 × 10^13^ m^−2^, and 5.95 × 10^12^ m^−2^. The variation pattern of $\rho^{GND}$ is shown in the GND density plot, and $\rho^{GND}$ decreases rapidly after the temperature drops to 1000 °C.

#### 3.1.3. Recrystallization Behavior

**Figure 12:**
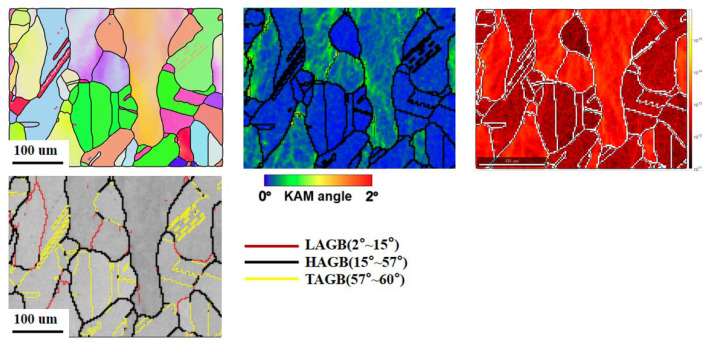
Additive manufacturing of 316L recrystallized at 1000 °C.

Smith et al. [27] found that the solidification substructure significantly influences dislocation mobility, requiring more power to achieve the same level of recrystallization in DED 304L compared to forged 304L, which requires higher temperatures or longer durations. As shown in Figure 12, after 1 h of heat treatment at 1000 °C, approximately 50% recrystallization was observed, indicating that static recrystallization had begun. Similarly, Herrera et al. [28] discovered that recrystallization in cold-rolled 316L stainless steel occurred at around 600 °C after 1 h of heat treatment, suggesting that DED materials are more thermally stable. Like cold-rolled stock, DED-treated materials in this temperature range exhibit excellent strength and ductility. At moderate annealing temperatures (400–800 °C), the yield strength decreases while tensile ductility increases, highlighting a significant trade-off between hardening and softening. After 60 min at 800 °C, the dislocations are no longer retained by the cellular walls, due to the homogeneous redistribution of elements within the solidification cells [8]. At higher annealing temperatures (>1000 °C), pronounced recrystallization was observed, resulting in mechanical properties similar to those of conventional 316L. The dislocation density dropped to approximately 1.33 × 10^13^ m^−2^, while the volume percentage of recrystallized grains increased rapidly.

**Figure 13:**
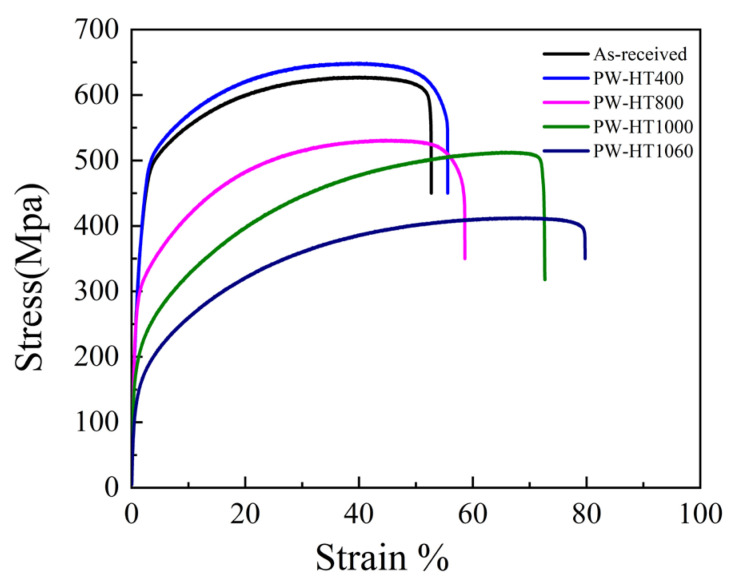
Engineering stress–strain curves for PW mode.

Figure 13 shows the engineering stress–strain curves for the samples, specifically illustrating the tensile behavior of DED samples subjected to different annealing processes. As-received PW samples exhibited a yield strength of 500 MPa, which decreased to 184.7 MPa after annealing at 1060 °C. For comparison, the typical yield strength of 316L stainless steel is around 160 MPa. The as-received samples demonstrated superior yield strength, likely due to the gradual disappearance of solidification microstructure cells at temperatures above 800 °C and the onset of recrystallization.

### 3.2. Surface Displacements and Residual Stresses

This section includes representations of surface displacements and residual stresses normal to the substrate’s cut surface.

#### 3.2.1. Surface Displacements

**Figure 14:**
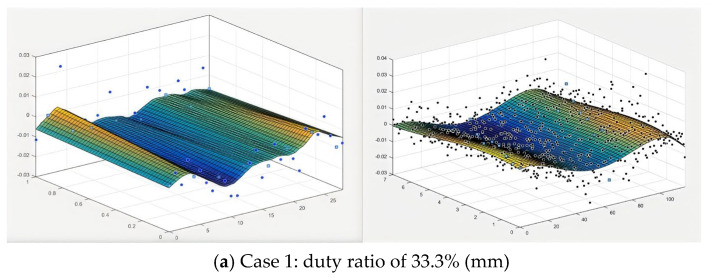

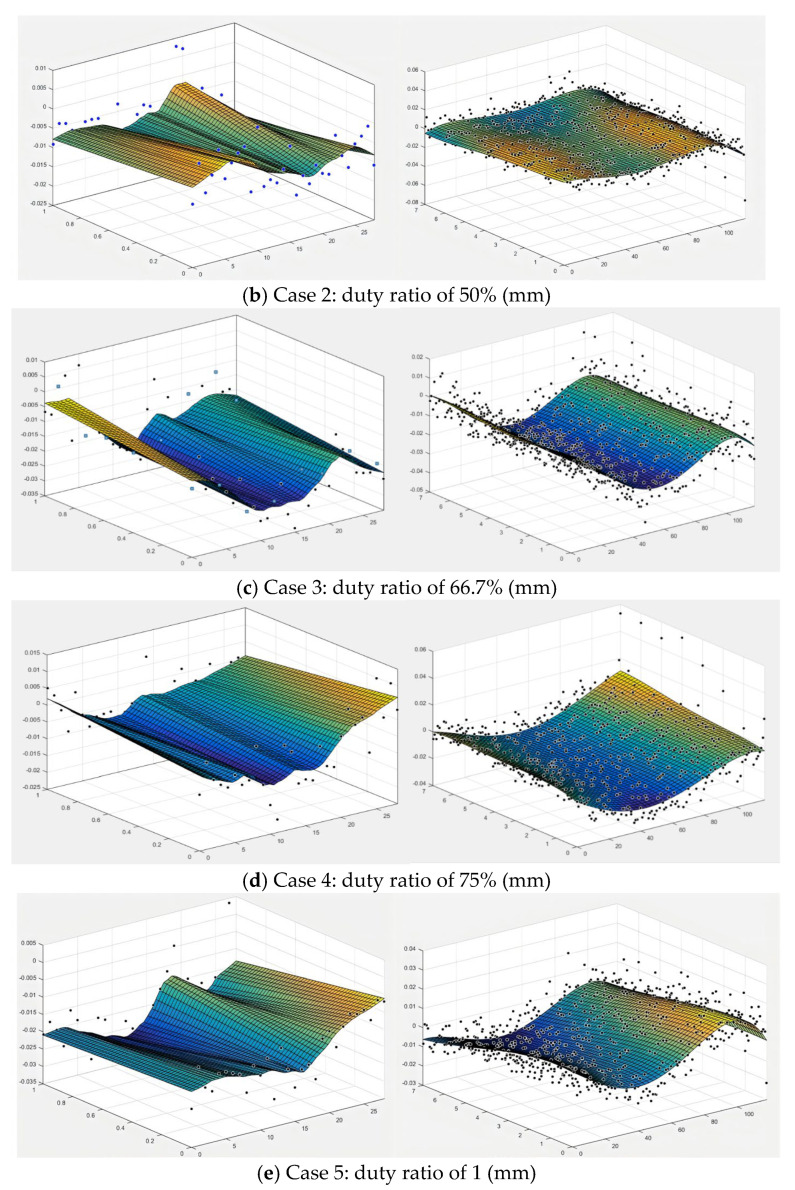
Displacement data in longitudinal direction. Left: low-resolution area (2-7 mm below the top surface); right: high-resolution area (0–2 mm below the top surface). (**a**) Case 1; (**b**) Case 2; (**c**) Case 3; (**d**) Case 4; (**e**) Case 5 under same pulse frequency of 10 Hz.

**Figure 15:**
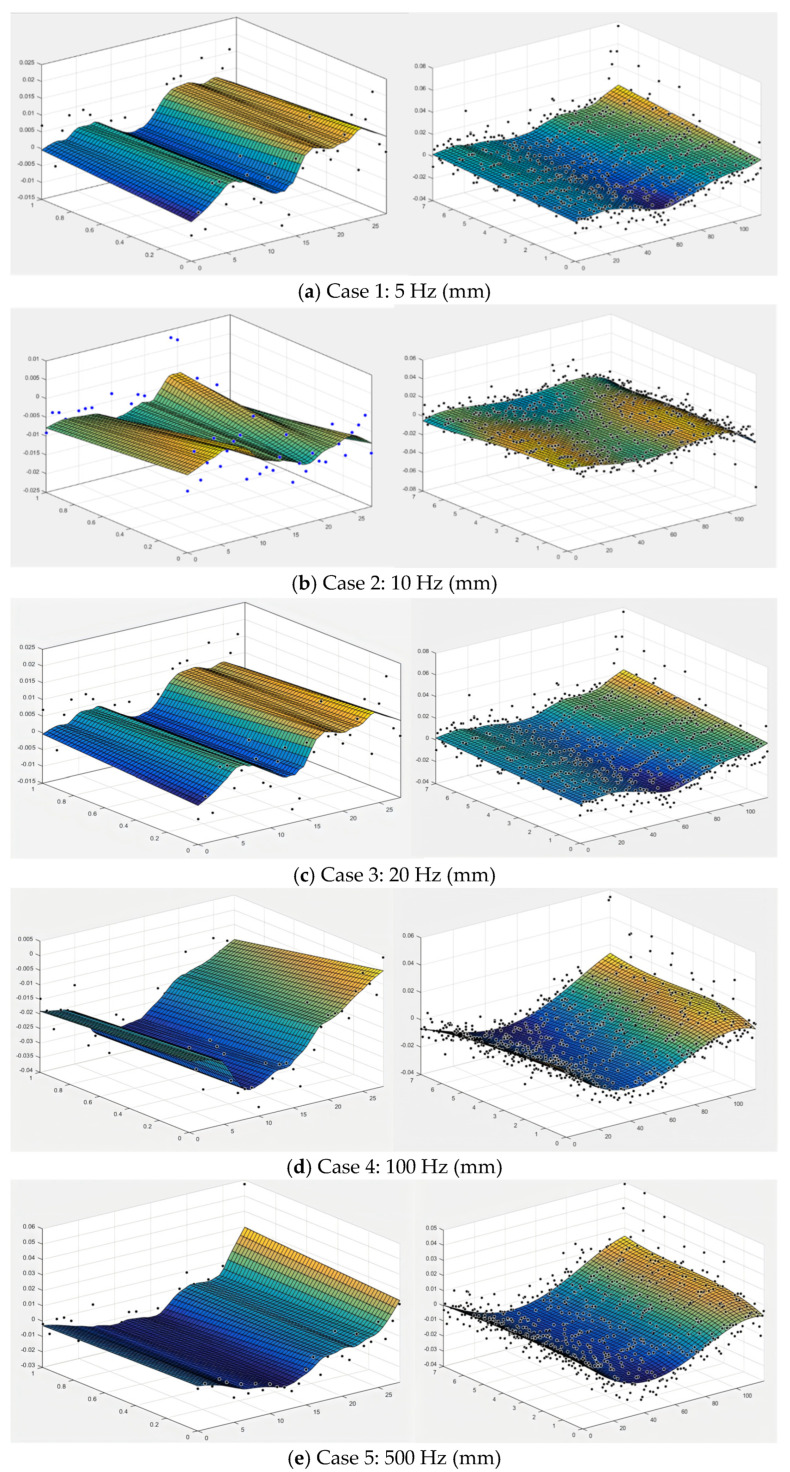
Displacement data in longitudinal direction. Left: low-resolution area (2-7 mm below the top surface); right: high-resolution area (0-2 mm below the top surface) under various pulse frequencies.

The results from the contour method provided 2D maps of the substrate surface displacements in the longitudinal direction under the same peak power for PW laser modes, as shown in Figure 14 and Figure 15, for different duty ratios and frequencies. The rapid temperature changes in the heat-affected zone (HAZ) during the laser deposition process can lead to the formation and accumulation of large gradient stresses. To capture these abrupt stress changes, two resolution levels were applied across the entire cross-sectional contour, as described in Section 3.2.1. The results exhibit a U-shaped distribution, with the sides rising and the middle concave. The peak-to-trough range for each plate is approximately 60 um. It is also worth noting that the edges of the samples appear concave, likely due to unavoidable data errors and measurement noise [30,31]. These errors and noise were subsequently filtered from the measured surface contour by fitting the data to a smooth analytical surface. The darker the color of the cloud image, the more intense the deformation. The dot represents the change in displacement. Therefore, under the same conditions, the smaller the total deformation of the sample with a duty ratio of 50% and under the same conditions, the smaller the total deformation of the sample at moderate laser frequencies (100 Hz).

#### 3.2.2. Residual Stress and Residual Strain

**Figure 16:**
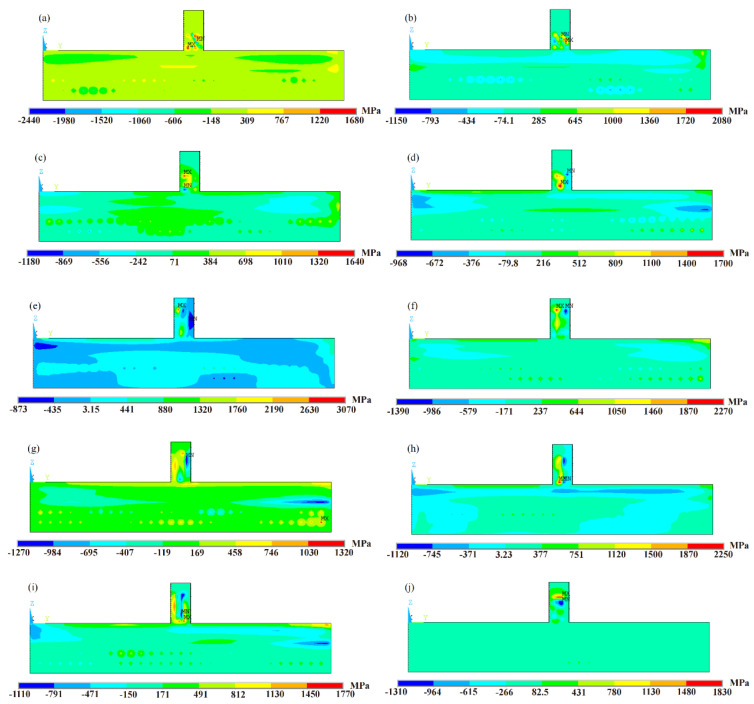
Two-dimensional mapping of the vertical residual stress ($\sigma_{z}$): (**a**) Case 1; (**b**) Case 2; (**c**) Case 3; (**d**) Case 4; (**e**) Case 5; (**f**) Case 6; (**g**) Case 7; (**h**) Case 8; (**i**) Case 9; (**j**) Case 10.

On six samples, the contour approach was used to analyze the residual stress in the substrate. The results are shown as contour maps in Figure 16. Consistent with the stress balance at a free surface, the longitudinal residual stress distribution near the substrate top displays a parabolic pattern in the horizontal direction, with tensile stress at the center and compressive stress at both ends. In the heat-affected zone (HAZ), the longitudinal residual stress changes dramatically with depth, especially at the substrate-deposited material junction (about half a millimeter from the substrate’s top). In this area, you can find the maximum tensile stress; from there, it changes to increasingly compressive values as it approaches the bottom of the substrate. The cooling shrinkage and thermal expansion that take place in the HAZ as a result of the laser deposition process are responsible for this behavior.

**Figure 17:**
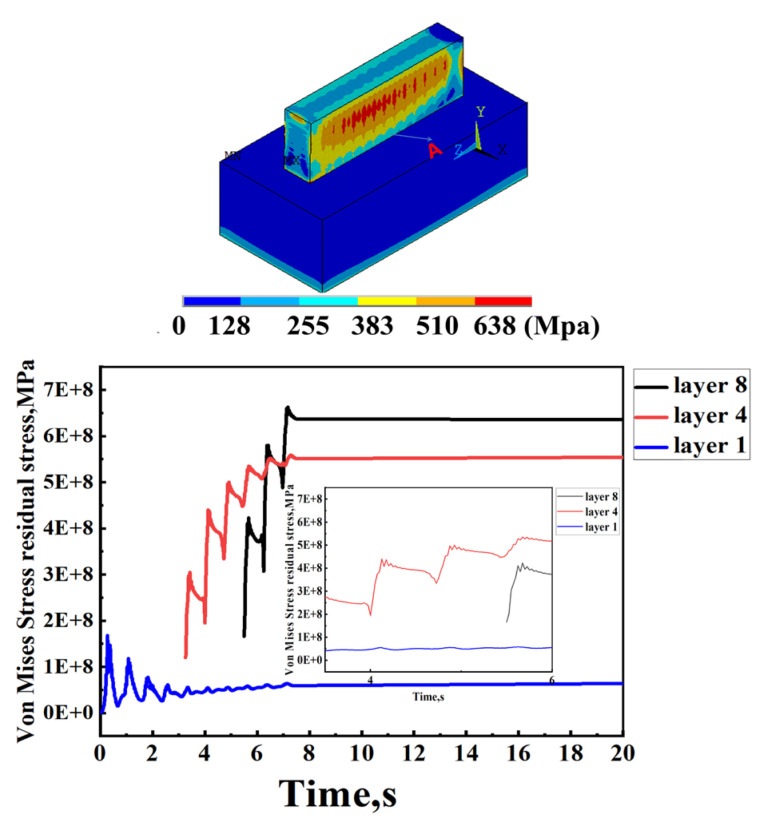
Evolution of Von Mises stress versus cladding (t).

The stress fluctuations in the lower levels are mostly stable, as illustrated in Figure 17, with the exception of the top few layers, which display local variances, the position of point A is the midpoint of the central section. This means that comparable physical effects emerge from the assumptions made here. All following discussions in this section assume one-layer melting for the sake of clarity and to provide deeper insight into the product-building process. Layers 1, 4, and 8 are added using this assumption in Figure 17. It has been noted that the residual tension in the topmost layer is reduced by adding more layers. This occurs because the layers below the one that was first deposited become thicker and more resistant to distortion with each successive layer. In a study on residual stress in AISI 18 Maraging Steel, Casavola et al. [32] also found comparable results.

**Figure 18:**
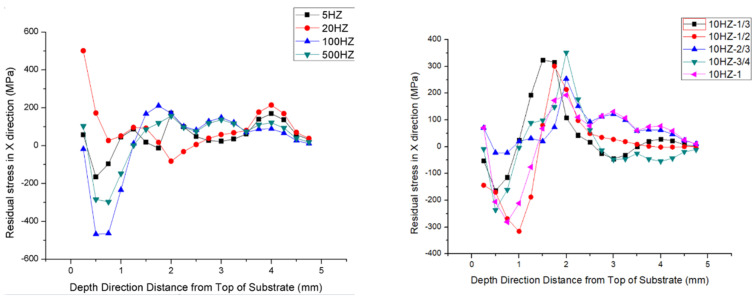
The longitudinal residual stress ($\sigma_{x}$) distribution along the vertical direction of the substrate on the mid-line of the cross-section.

At varying depths from the substrate’s top, Figure 18 illustrates the maximum longitudinal residual stress in the heat-affected zone of specimens with pulse frequencies of 5 Hz, 20 Hz, 100 Hz, and 500 Hz, and a duty ratio of 1/2. At 500 Hz, the residual stress reaches a value of approximately 552 MPa or higher, which is slightly greater than the yield strength of the base material. This is due to the strain-hardening effect of the material and the triaxial stress state of the residual stress. Moreover, the high laser power leads to a high energy input, which reduces the yield strength by converting the material from brittle to ductile. The vaporization of alloying elements with lower boiling points can be a consequence of the melting process’s instability, which influences the mechanical properties of the produced parts and the material composition. The components exhibit substantial residual stresses as a result of these factors [27]. Consequently, the longitudinal residual stress at the joint in the heat-affected zone increases as the laser power increases for PW samples.

The maximum longitudinal residual stress in the heat-affected zone is depicted in Figure 18 for specimens with varying depths from the top of the substrate and different duty ratios of 1/3, 1/2, 2/3, 3/4, and 1. The pulse frequency is 10 Hz. In both CW and PW modes, a stress gradient of 150 to 200 MPa is observed at the junction between the substrate and the deposited materials under the same peak laser power. The maximum longitudinal residual stress is observed at the junction between the substrate and deposited materials in the heat-affected zone for samples in the PW mode with a 3/4 duty ratio, as illustrated in Figure 18. The temperature history curve of the molten pool center in PW modes can be employed to elucidate this phenomenon [33,34,35]. Additionally, the reduced temperature gradient at the junction between the substrate and deposited materials is a result of the periodic thermal expansion and cooling contraction caused by the cyclical switching of light in PW mode [36,37].

**Figure 19:**
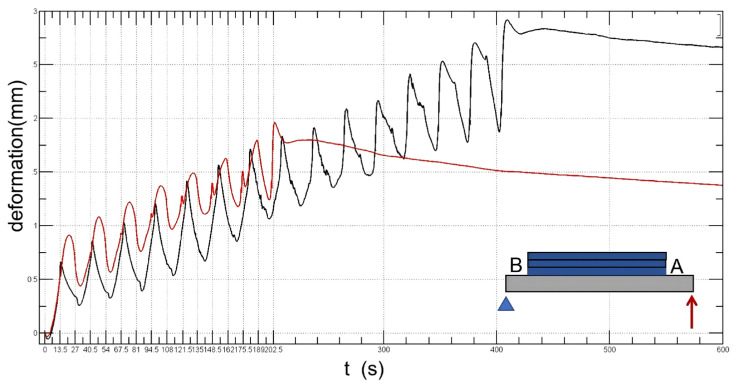
The influence of different scan paths on deformation. The black line represents 15 unidirectional printing layers, and the red one represents 15 bidirectional printing layers.

**Figure 20:**
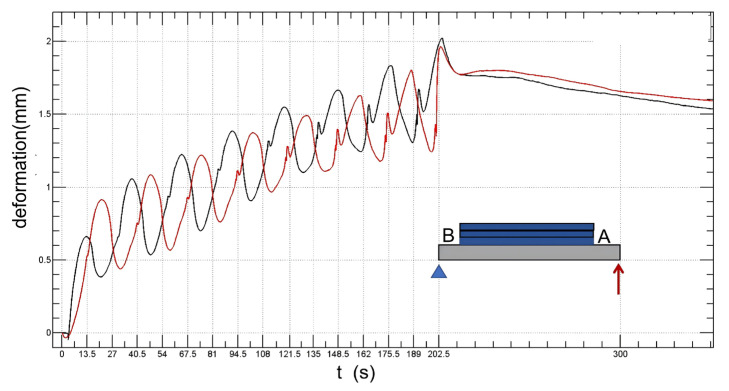
The effect of different scanning points on the amount of deformation. The black line indicates the light from the fixed end B, and the red line indicates light from the free end point A.

The deformation points of the substrate measured by the displacement sensor are shown in Figure 19 and Figure 20. Figure 19 illustrates the influence of different scan paths on deformation. The black line represents 15 unidirectional printing layers, and the red one represents 15 bidirectional printing layers. The results show that the deformation value of the substrate in bidirectional scanning is much smaller than that in unidirectional scanning. The residual strain in unidirectional scanning is 1.6 mm, and the residual strain in bidirectional scanning is as high as 5.4 mm. On the other hand, Figure 20 illustrates the effect of different scanning points on the amount of deformation. The black line indicates the light from the fixed end B, and the red line indicates light from the free end point A. The results show that the effect of the initial light spot on the residual strain is at about 1.6 mm.

## 4. Conclusions

The primary conclusions of this investigation are outlined as follows: The phase stability and microstructure of PW SS316L stainless steel fabricated by DED were examined in relation to the effects of annealing at varying temperatures. The corresponding changes in mechanical properties under tensile loading were also analyzed. The findings suggest that the cell size increases as the annealing temperature increases, culminating in the disappearance of the microstructure at temperatures exceeding 1000 °C. In the interim, the specimens’ strength diminishes as the annealing temperature increases. Furthermore, residual stress tests conducted at a variety of pulse frequencies and duty cycles demonstrated that residual stress is effectively reduced by 50% at moderate laser frequencies (100 Hz) and effectively reduced by 18.6% with moderate laser duty cycles (1/2).

## Figures and Tables

**Table 1:** Element composition.

	Fe	Cr	Mn	Si	Cu	Mo	Nb	Ni
Percentage	Bal.	16.92	0.54%	3%	7.2%	3%	5%	11.66%

**Table 2:** Parameters for laser processing.

Case	Mode	Power (W)	Scanning Speed (mm/s)	Rate from Powder Feeding (g/min)	Number of Layers	Pulse Frequency (Hz)	Duty Ratio
1	PW	500	8	8.3	20	10	33.3%
2	PW	500	8	8.3	20	10	50%
3	PW	500	8	8.3	20	10	66.7%
4	PW	550	8	8.3	20	10	75%
5	CW	500	8	8.3	20	10	1
6	PW	500	8	8.3	20	5	50%
7	PW	500	8	8.3	20	20	50%
8	PW	500	8	8.3	20	100	50%
9	PW	500	8	8.3	20	500	50%

## Data Availability

Dataset available on request from the authors.
